# Alpha-Thalassemia in Southern Italy: Characterization of Five New Deletions Removing the Alpha-Globin Gene Cluster

**DOI:** 10.3390/ijms24032577

**Published:** 2023-01-30

**Authors:** Giovanna Cardiero, Gennaro Musollino, Romeo Prezioso, Vincenzo Nigro, Giuseppina Lacerra

**Affiliations:** 1 Institute of Genetics and Biophysics “Adriano Buzzati Traverso” (IGB-ABT, CNR), National Research Council, 80131 Napoli, Italy; 2 Department of Precision Medicine, University of Campania L. Vanvitelli, 80138 Napoli, Italy; 3 Telethon Institute of Genetics and Medicine (TIGEM), 80078 Pozzuoli, Italy

**Keywords:** alpha-thalassemia, MLPA, qRT-PCR, deletion breakpoint characterization, --Sciacca, --FG, --PA, --Puglia, --AG

## Abstract

α-thalassemia is characterized in about 80% of cases by deletions generated by the presence of duplications and interspersed repeated sequences in the α-globin gene cluster. In a project on the molecular basis of α-thalassemia in Southern Italy, we identified six families, showing an absence of the most common deletions, and normal α-globin gene sequences. Multiplex Ligation-dependent Probe Amplification (MLPA), qRT-PCR, and the sequencing of long-range PCR amplicon have been used for the identification and characterization of new deletions. MLPA analysis for the identification of α- and β-globin rearrangement revealed the presence of five new α-thalassemia deletions. The set-up of qRT-PCR allowed us to delimit the extent of the deletions ranging from about 10 kb to more than 250 kb, two of them being of the telomeric type. The long-range PCR generated a specific anomalous fragment in three deletions, and only several unspecific bands in the other two deletions. The sequencing of the anomalous amplicons revealed the breakpoints of two deletions: the --PA, 34 kb long, identified in two families, and the telomeric --AG, 274 kb long. The anomalous fragment containing the breakpoint of the deletion --FG was partially sequenced, and it was not possible to identify the breakpoints due to the presence of several repetitive Alu sequences. The analysis of the breakpoint regions of the --Sciacca and --Puglia, respectively, are about 10 and 165 kb long, and revealed the presence of repeats that most likely impaired the amplification of a specific fragment for the identification of the breakpoint. MLPA, in association with qRT-PCR and long-range PCR, is a good approach for the identification and molecular characterization of rare or new deletions. Breakpoint analysis confirms that Alu sequences play an important role in favoring unequal crossing-over. Southern Italy shows considerable genetic heterogeneity, as expected with its central position in the Mediterranean basin, favoring migratory flows.

## 1. Introduction

α-thalassemia is a genetic autosomal recessive disorder, characterized by the partial (α+) or total (α^0^) reduction of the α-globin chain synthesis. The World Health Organization (WHO) has estimated that more than 20% of the world population is heterozygous for an α-thalassemia allele [1]. A higher number of α-thalassemia heterozygotes is described in the Mediterranean countries, South-East Asia, Africa, the Middle East, and in the Indian subcontinent [2].

The synthesis of the globin chains is regulated by four α-globin genes (αα/αα), two for each copy of chromosome 16. For this reason, there are different genotypes of α-thalassemia depending on the number of alleles involved, and consequently the carriers show high phenotypic heterogeneity [3].

The α-globin gene cluster contains the duplicated α-globin genes (*α1–α2*), the embryonic gene (*ζ2*), and the pseudogenes (*ψζ1, ψα2*, and *ψα1*); it is located in a DNA region rich in pairs of nitrogenous bases (guanine–cytosine), and of Alu-family repeated sequences. Moreover, the α1 and α2 genes are enclosed into highly repetitive regions (X, Y, and Z), which favor reciprocal recombination in the chromosome, but can also promote the wrong alignment which could lead to unequal crossing-over, resulting in the deletion of one or both the α-globin genes.

In the regulation of the α-like globin genes, four Multispecies Conserved Sequences Regions (MCS-R) are involved, called MCS-R1 to -R4, located 25–65 kb upstream of the α-globin genes. Out of these four elements, only MCS-R2 has been shown to be essential for the α-globin expression [4].

In the α-thalassemia, point mutations represent about the 10–20% of the total alterations [5,6], currently found with increasing frequencies due to the indirect or direct methodologies able to identify the point mutations [7], unlike β-thalassemia for which the most frequent molecular alterations are point mutations. In Southern Italy, the frequencies of the α-thalassemia point mutations represent about the 25% of the total alterations [8].

The most frequent molecular alteration in α-thalassemia is represented by deletions of various extension, with the loss of the regulative sequences or of one or both the α-globin genes on the same chromosome [2]. The hematological characteristics, which depend on the number of deleted α-globin genes, are often evident and consist of marked microcythemia, hypochromia, and polyglobulia [9].

About seventeen α-thalassemia deletions have been found in the Mediterranean area. --MED I, --MED II, -(α)^20.5^, and -α^3.7^ have shown high frequencies in Mediterranean populations; -α^3.7^, --MED I, -(α)^20.5^, --CAL, -α^4.2^, and -α^5.2^ have been found very frequently in Italy while the (α)α^5.3^ and --Campania have been identified in few families from Campania; --MA, --CL, --SPAN, --CANT, and --CAL have been found in the Spanish population; and –BGS and -α^5.2^ have been found in the Greek population. --MED II was identified, also, in a few Turkish families [2,9,10,11,12].

Due to the high frequency of deletions affecting the α-globin cluster, it is necessary to identify the carriers in Italy and in the Mediterranean area, in order to prevent the most severe forms, such as Hb H disease and Hb Bart’s.

In the present study, we analyzed well-diagnosed hematological α-thalassemia carriers, negative in gap-PCR, Denaturing Gradient Gel Electrophoresis (DGGE), and sequencing analysis, for deletions in the α-globin gene cluster by MLPA, qRT-PCR, and long-range PCR. Five new α-thalassemia deletions have been identified and characterized.

## 2. Results

All the carriers of the deletions reported below showed the absence of the most frequent deletions present in the Mediterranean area and the normal sequence of the *HBA1* and *HBA2* genes.

### 2.1. Family 1, --Sciacca

**Figure 1:**
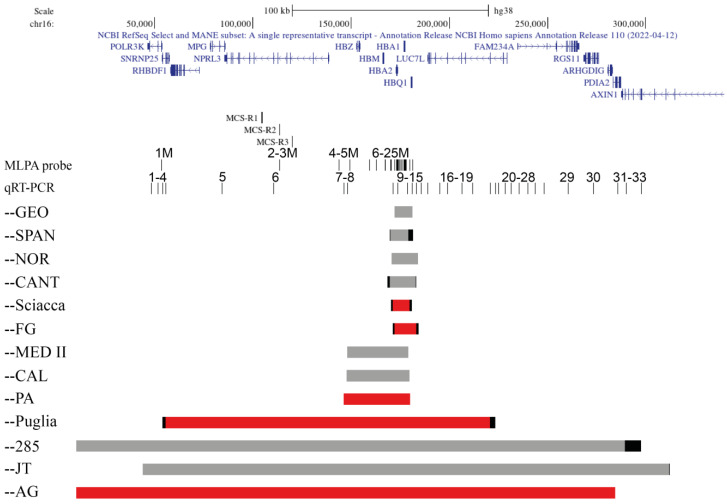
Scheme of the telomeric region on chromosome 16 containing the α-globin gene cluster. In the upper part are reported the genes and the MCS-R, then the location of the MLPA probes (indicated with numbers followed by the letter M) and of the quantitative real-time PCR fragments (indicated with numbers). The five new deletions (--Sciacca, --FG, --PA, --Puglia, and --AG) are indicated in red, the deletions already described are indicated in grey, and the undefined region are in black. Four known deletions --GEO, --SPAN, --NOR, and --CANT are compared with the new deletions --Sciacca and --FG. --PA is compared with two known deletions --MED II and --CAL. The --AG is compared with the --285 and the --JT deletions.

**Figure 2:**
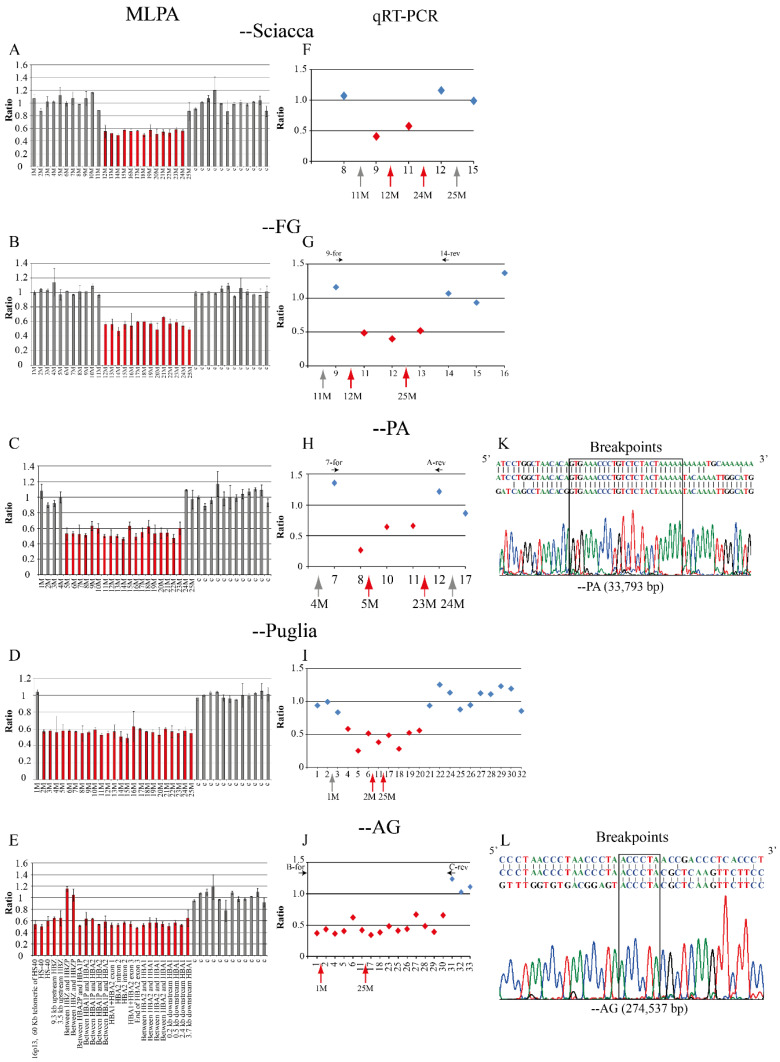
Molecular characterization of the five new alpha-thalassemia deletions. (**A**–**E**) MLPA analysis of five new deletions. Comparison between the value obtained from the proband examined and normal control (*y*-axis). The position of the different probes on the α -globin gene cluster is indicated on the *x*-axis. In red, the probes with half-dose; in gray, the probes with normal dosage. (**F**–**J**) Quantitative real-time PCR analysis of 33 probes (primers are reported in Table S2) posed in regions not tested by MLPA: red diamonds show fragments in half-dose; blue diamonds show normal dosage. The localization of the MLPA probes is indicated by the arrows. (**K**,**L**) Sequencing of the long-range gap-PCR fragments. In the middle has been reported the breakpoint of the deletion, above and below, respectively, the regions at 5′ and 3′. The new deletions most likely originated from events of non-homologous recombination mediated by short sequences (highlighted with a box), common to both the extremities and most likely causing the formation of loops. The HGVS code of the --PA is NC_000016.10:g.(146281_146304)_(180074_180097)del33793 and the HGVS code of the --AG is NC_000016.10:g.10001_(284537_284542)del274537.

The proband was a man of Sciacca, Sicily, showing microcythemia and polyglobulia (MCV 70.8 fL, MCH 22.2 pg), with normal levels of HbA2 (2.5%) and HbF (1.9%). The iron balance was not performed (Table 1). The MLPA analysis highlighted a deletion removing 13/25 probes leaving the 5′ of the α-globin gene cluster (Figure 1 and Figure 2A). The deletion was named --Sciacca (Figure 1). The qRT-PCR analysis of five probes showed that two of them (9 and 11) were deleted (Figure 2F). This method allowed us to reduce the extension of the deletion detected by MLPA, to a length between 8684 bp and 10,800 bp. In the undefined zone at the 5′ breakpoint among the MPLA probe 11M and the qRT-PCR probe 9, 776 bp long, there is one Sine/Alu element present, while in the indefinite zone at the 3′ breakpoint, between the MPLA probe 24M and the qRT-PCR probe 12, 1377 bp long, two Sine/Alu elements and one Sine/Mir element are present (Figure S2A). It was not possible to design additional probes for the qRT-PCR analysis and to amplify a fragment by long-range PCR, due to the high repetitiveness of sequences present at the 3′ and 5′ regions of the breakpoints.

### 2.2. Family 2, --FG

**Figure 3:**
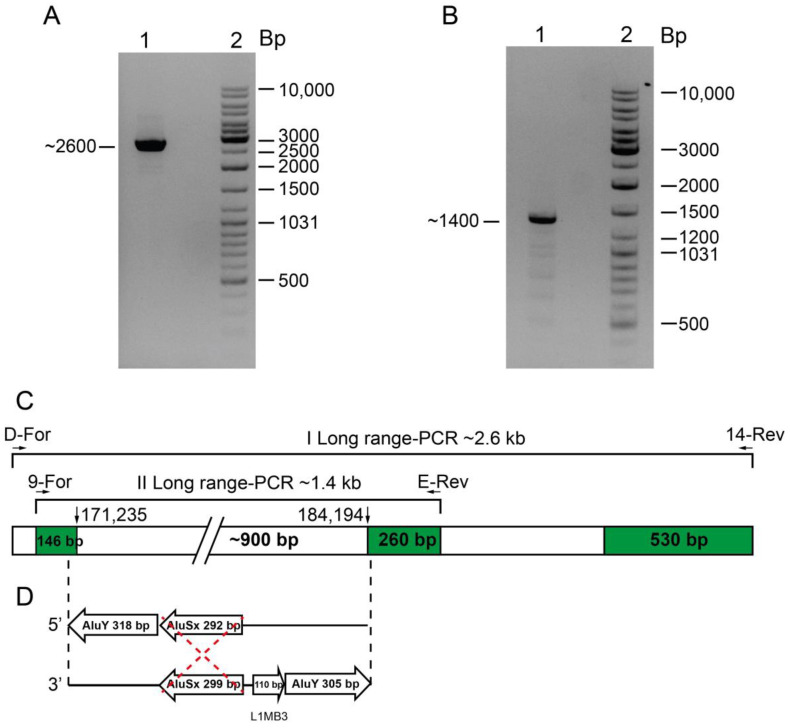
Molecular characterization of the --FG deletion. (**A**,**B**) Product of the long-range GAP-PCR separated on new-Sieve agarose gel. Line 1: carrier of the --FG deletion; line 2: ladder. (**C**) Scheme of the sequencing analysis of the amplicons. In the upper part have been reported the oligos and the length of the amplicons. Green boxes indicate the sequenced regions, white boxes the un-sequenced regions. The number close to the vertical arrow refer to the last sequenced positions on the chromosome. (**D**) Probable breakpoint. The boxes with arrows indicate the repeated sequences and the orientation located, respectively, at 5′ and 3′ of the breakpoint. The red dotted lines indicate the possible recombinant event.

Family 2 was composed of two parents and three sons, from Foggia. The three brothers showed microcytic and hypochromic anemia (MCV 60.5–64.2fL, MCH 18.4–19.0 pg), with the presence of hemoglobin H (Hb H 6.7–9.9%) (Table 1). These phenotypes suggested a large deficit of α-globin chains. The mother showed a similar phenotype, while a milder phenotype was seen in the father. The molecular screening showed the presence of the -α^3.7^ deletion in the father and sons, but the absence of large deletions or point mutations. The MLPA analysis revealed, in addition to the common -α^3.7^ deletion, a new deletion of about 10 kb, present in the mother and sons. In particular, the MLPA analysis revealed reduced signals in 14/25 probes, from the probe 12M to the probe 25M (Figure 1 and Figure 2B). The breakpoint region was narrowed by qRT-PCR: one breakpoint was localized between the qRT-PCR probe 9 to the MLPA probe 12M, and the other was localized between qRT-PCR probes 13 and 14 (Figure 2G). The long-range gap-PCR, performed using the oligonucleotides D-for and 14-rev, amplified an anomalous fragment of about 2600 bp. The sequences analysis using the oligo 9-for at the 5′breakpoint showed a normal sequence of 146 bp, till position 171,235, inside an Alu Y sequence. The fragment of 2600 bp at the 3′ end was sequenced with the oligo 14-rev showing a normal sequence of about 500 bp (Figure 3). Despite the use of different oligos, further sequencing was not possible, most likely because, in the region, there are two Alu elements (co-ordinate 171,206–171,523 and 171,531–171,822) (Figure 3). A new long-range PCR was performed using the primers E-rev, designed in the upstream 3′ region, with the primer 9-for, generating a fragment of about 1400 bp (Figure 3). This fragment was sequenced with the E-rev probe but the sequence was interrupted, however, after 248bp, at position 184,194, probably because in the contiguous area are present a strand of A and two Alu and one Line element (co-ordinate 183,449–183,747, 183,780–183,889, and 183,901–184,205, respectively). For this reason, it was not possible to sequence the fragment of about 900 bp to identify the breakpoint located between the co-ordinate 171,235 and 184,194 (Figure 3).

The analysis of the breakpoint’s regions indicated the presence of five Alu sequences, respectively, two in the 5′ breakpoint and three in the 3′ breakpoint, as reported in Figure 3. The analysis of the type and distance between the Alu sequences suggested that most likely an unequal crossing-over could have occurred between the SINE Alu SX sequences present both in the 5′ and in the 3′ region, generating the deletion of about 12,000 bp and the formation of a region of about 900 bp, corresponding to the distance between the two sequenced regions at 5′ and 3′ of the 1400 bp fragment, obtained by long-range PCR.

### 2.3. Family 3 and 4, --PA

The deletion --PA was found in five members of two unrelated family from Palermo, Southern Italy.

Family 3 was composed of four people, including two children (I.1, II.1, III.1, and III.2). Three of them showed a reduction in the MCV (range of 63.0–67.0 fL) and MCH (range of 20.1–20.9 pg), with normal levels of HbA2 (range of 2.4–2.6%). Hematological parameters suggested the presence of an α-globin gene cluster deletion.

Family 4 was composed of three adults. The patient I.1 and the son II.1 showed hypochromic and microcytic red blood cells (MCV 70.0 fL, MCH 25.0, 29.5 pg), with a normal level of HbA2 (2.6%) for Patient I.1, while Patient II.1 had an increased level of HbA2 (6.3%). Sequencing of the β-globin gene showed the presence of the β-thalassemia mutation IVS-II-745 (C>G) (Table 1). The other son II.2 had normal hematological indices, and a normal level of HbA2 (3.0%).

The deletion --PA was identified by MLPA analysis: 19/25 probes had a reduction of signals, specifically from the probe 5M up to the probe 23M (Figure 1 and Figure 2C). The analysis by qRT-PCR of six probes showed that three of them were deleted (8, 10, and 11). The possible breakpoint region was localized at 5′ between the qRT-PCR probes 7 and 8, and at 3′ between the MLPA probe 23M and the qRT-PCR probe 12 (Figure 2H). The definition of the breakpoints was obtained by amplifying a fragment by long-range PCR with oligos 7-for (co-ordinate 146,054–146,073) and A-rev (co-ordinate 180,344–180,321) that generated an amplicon of 501 bp (Table S2). The sequencing of this fragment identified in the breakpoints a sequence of 24 bases common to the 5′ and 3′ regions that could have facilitated the recombination. The deletion is localized at 5′ at position 146,281–146,304 and at 3′ at position 180,074–180,097 (Figure 2K). The length of the novel --PA deletion was 33,793 bp.

### 2.4. Family 5, --Puglia

The proband was a woman from Foggia, Southern Italy, showing microcytic and hypochromic anemia (MCV 73.0 fL, MCH 22.8 pg), with a normal iron balance and normal HbA2 (2.7%) and HbF (0.3%) (Table 1). The MLPA analysis of the α-globin gene cluster showed that 24/25 probes, from probes 2M to 25M, had a decreased intensity, suggesting the presence of a long deletion removing all the α-globin gene cluster (Figure 1 and Figure 2D). The deletion was called --Puglia based on the geographic origin of the carriers. To delimit the deleted regions, the qRT-PCR analysis of 21 probes was performed, showing that the region from the probe n. 4 up to the probe n. 20 was deleted, while the first three and the final 10 probes were still present (Figure 2I). In the undefined regions, at the 5′ breakpoint between the probes 3 and 4, 1417 bp long, are present two Sine/Alu and three short Mer96b elements, while in the indefinite zone at the 3′ breakpoint, between the probes 20 and 21, 2609 bp long, are present six Alu elements (Figure S2B,C). The long-range PCR gave rise to several unspecific fragment, most likely due to the presence of several repetitive elements, and for this reason, it was not possible to perform the sequencing analysis to identify the breakpoint of the --Puglia deletion. The 5′ undefined region is between position 54,016 up to 55,432, and the 3′ undefined region is between position 220,780 up to 223,388, with the deletion showing an extension of at least 165,347 up to 169,373 bp.

### 2.5. Family 6, --AG

The new deletion called --AG was found in a family with two children. The father and sons showed microcytic and hypochromic anemia, with reduced mean corpuscular volume (MCV) 66.2–68.0–70.8 fL, and mean corpuscular hemoglobin (MCH) 21.0–22.7–21.1 pg, but a normal level of HbA2 (2.5–2.8%), while the mother had normal hematologic parameters (Table 1).

The MLPA analysis showed reduced signals of 23/25 probes, indicating the presence of a very long deletion including the telomeric region. The MLPA probes n. 6 and n. 7 showed normal levels in the son due to the presence of a duplication of the zeta region as observed also in the mother (Figure S1B). The father showed the deletion of all the MLPA probes and absence of the duplicated zeta region. (Figure S1A). By qRT-PCR were analyzed regions not tested by MLPA: out of the 18 probes, 15 were deleted, and nine of them allowed the further extension of the 3′ delete region (Figure 1 and Figure 2J). The qRT-PCR analysis narrowed the breakpoint region: one breakpoint was localized in the telomeric region, and the other was located between the qRT-PCR probes 30 and 31 (Figure 2J).

The long-range gap-PCR using the oligos B-for and C-rev generated a fragment of 522 bp (Table S2); its sequence showed the presence of a 6mer “ACCCTA” common to the 5′ and 3′ breakpoints and indicated that the 5′ breakpoint was localized in the telomeric region, and the 3′ breakpoint at position 284,537-284,542 (Figure 2L). The length of this novel deletion, named --AG, was 274,537 bp. We also set up a specific gap-PCR protocol, with internal control, for a rapid identification of the heterozygotes.

## 3. Discussion

In the present paper, we report five new deletions removing a different part of the α-globin gene cluster characterized by the loss of functions of both the α-globin genes. Out of them, three deletions involved only the α-globin gene cluster and the other two are longer telomeric deletions removing the cluster and the regulative elements. The combination of several approaches (MLPA, qRT-PCR, long-range PCR, and sequencing) gave us the chance to reduce significantly the boundary breakpoint regions, and for two of them, the --AG and the --PA, to define the breakpoints.

### 3.1. Deletion Removing the α-Globin Gene Cluster

Out of the three deletions removing only the α-globin gene cluster, two are about 10 kb long (--Sciacca and --FG) and the third one is 36 kb long (--PA).

The --Sciacca and the --FG showed very similar MLPA patterns both starting from probe 12 M but ending, respectively, at probe 24 M and 25 M. The RT-qPCR analysis allowed us to define more precisely the position and extension of the deletions.

The --Sciacca showed an extension between 8697 bp up to 10,800 bp and removed the α2- and α1-globin genes, and the 3′ undefined region could show the deletion also of the *HBQ1* gene. Two undefined regions were present: the 5′ region contains a Sine/Alu element and the 3′ region two Sine/Alu and one Sine/Mir elements (Figure S2A). The presence of these repeated elements made it difficult to perform the long-range PCR protocol which produced several non-specific bands. The presence of repetitive elements could have favorited the unequal crossing-over event generating the deletion.

The second deletion named --FG was identified in a family with three patients showing Hb H disease. The --FG removes the α2- and α1-globin genes and the *HBQ1* gene. By long-range PCR was amplified a first fragment of about 2600 bp and its sequence analysis give us the opportunity to reduce the breakpoint region, amplifying a second shorter fragment of 1400 bp. The sequencing of this fragment was interrupted by the presence, respectively, of a strand of T (5′) and A (3′) bases. The breakpoint is located between the co-ordinate 171,235 and the co-ordinate 184,194.

In the same regions have been described four other deletions: the --Cant NC_000016.10:g.(168531_169756)_(182770_183028)del14497 kb [13], the --SPAN NC_000016.10:g.(169756_170100)_(179044_181595)del11839 kb [14], the --Geo (NG_000016.10:g.172001_181401del9401) [15], and the --NOR NC_000016.10:g.170694_184101del13408 [16]. The analysis of the position and extension of these known deletions indicated that they are located in different positions and that the --FG and the --Sciacca are new deletions with a breakpoint sited in the hot spot of recombination regions.

The third deletion removing the α-globin gene cluster is the --PA. The deletion was identified in two unrelated family from Palermo in a total of five carriers. A subject with a high level of Hb A2 (6.3%) showed also the presence of the β-thalassemia mutation IVS-II-745 C>G. The combination of the MLPA, RT-qPCR, and long-range PCR allowed us to define the breakpoints of the deletion that was 33,793 bp long NC_000016.10:g.(146281_146304)_(180074_180097)del33793.

In close proximity have been described two deletions, the --Med-II (NG_0000016.10:g.147936_179144del31208) also known as –DANE [17,18] and the --CAL (NG_0000016.10:g.147599_179800del32201) [19]. The definition of the breakpoint allowed us to ascertain that the --PA is a new deletion located in a region with a high rate of recombination.

The identification of the --PA in two families from Palermo for a total of five carriers suggests to look for this deletion in Sicilian patients negative for the gap-PCR screening of the most common deletions.

### 3.2. Deletions Removing the Regulative Elements and the α-Globin Cluster

Two telomeric deletions, the --AG and the –Puglia, were identified, respectively, in a family from Agrigento and a family from Puglia.

The --AG or NC_000016.10:g.10001_(284537_284542)del274537 is the longest (telomeric) deletion identified by us, spanning from the telomeric region in 5′ the genes *POLR3K*, *RHBDF1*, *MPG*, *NPRL3*, all the α-globin gene cluster, *LUC7L*, *FAM234A*, and *RGS11*, to the exon 2 of the *PDIA2* gene in the 3′. The *AXIN1* gene is not involved in the deletion.

The deletion --Puglia removes 24/25 of the MLPA and 8/21 of the qRT-PCR probes and shows an extension between 165 and 169 kb removing a region containing *MPG*, *NPRL3*, and all the α-globin gene cluster, to about 3/4 of the *LUC7L* gene. The undefined breakpoint regions (about 4 kb long) are characterized by the presence of several repetitive elements that could have favored the occurrence of the recombinant event. The deletion removes the α-globin gene starting at the 6340 bp 5′ of zeta to the 2500 bp 3′ of the α1 globin gene.

Several large telomeric deletions removing the α-globin gene cluster on the short arm of the human chromosome 16 (16p13.3) have been described, but as far as we know, none have been described in Italy. In the heterozygous state, the resulting phenotype consists of α-thalassemia for relatively short deletions (100 to 356 kb), while an α-thalassemia mental retardation syndrome (ATR-16 syndrome) is observed for larger deletions (>1 Mb) which generally include the 16p telomere [20,21]. In accordance with these data, the deletions --AG (274 kb) and --Puglia (165 up to 169 kb) are associated with the α^0^-thalassemia phenotype, and not to the α-thalassemia mental retardation syndrome (ATR-16 syndrome).

In close proximity of the --AG deletion have been described the --JT or NC_000016.10:g.(44035_44092)_(312033_312090)del268kb and a Chinese deletion of about 285 kb, the last one in the heterozygous condition showing marked scoliosis [2,21,22]. Few data are available on the --JT and its origin is not known. The --AG is shorter than the --285 and it is not associated with scoliosis. The --AG is longer than the --JT (268 kb) but positioned at 5′.

In Italy, Colosimo et al. (2011) performed an analysis in a cohort of 18 individuals suspected to have α-globin alterations, but no mutations using conventional methods, and in 5 Hb H patients where only the -α^3.7^ deletion was detected. The individuals were either of Mediterranean or of South-East Asian origin. In five individuals of various origins, Colosino et al. observed differently sized α^0^-thalassemia deletions ranging from the HS-40 regulatory region to the α-globin locus. Comparing the MLPA pattern of the five new deletions identified by us with that reported in the article by Colosimo et al. (2011), it emerges that four out of five deletions showed a position not compatible with that of our samples, and that sample 16 was a telomeric deletion removing chromosome 16 from the *PolR3k* gene to the *HBQ1* gene. The nationality of this sample is not specified [6].

Considering that the origin of the patient is not known, that the MLPA analysis stopped at about position 180 kb of the chromosome, and that the telomeric deleted region can be very long, there are no specific data indicating that could be the same deletion --AG identified by us.

From our data, it appears that --AG and --Puglia are the first telomeric deletions identified in Italy and in the Mediterranean basin.

## 4. Materials and Methods

### 4.1. Patients and Hematological Data

In a project on the molecular basis of α-thalassemia in Southern Italy, the thalassemia centers collaborating in this study selected families/single patient showing an MCV lower than 80 fL associated with normal Hb A2 and iron status. We report the results of the study on 14 carriers belonging to 6 unrelated families living in Sicilia and Puglia. Ethical approval of the protocol was obtained from the Comitato Etico Università Federico II (307/2016; 225/2019).

The hematological data (Table 1), serum iron, ferritin, and total and indirect bilirubin were obtained by standard methods in the collaborating hospitals. Hb level measurement was performed by the cation exchange high-performance liquid chromatography (HPLC) (Bio-Rad, Diamat, or Variant System Hercules, CA, USA). The research activities were carried out inspired by the principles of quality, and by developing guidelines for the protocols to increase the reliability and reproducibility of the results [23,24].

### 4.2. DNA Analysis

DNA extraction from white blood cells was performed by salting-out method. The presence of the most common deletions frequently found in the Mediterranean basin (-α^3.7^, -α^4.2^, -(α)^20.5^, --MED, --CAL) were tested by means of gap-PCR protocols. The multiplex Amplification Refractory Mutation System (ARMS), DGGE, Denaturing HPLC (DHPLC), and sequencing analysis of the *HBA1* and *HBA2* globin genes were performed to exclude the presence of point mutations [25,26].

### 4.3. MLPA Analysis

MLPA was carried out using the Salsa MLPA Kit P140B HBA and SALSA MLPA KIT P102 HBB (MRC-Holland, Amsterdam, The Netherlands) according to the manufacturer’s instructions and as already reported [1,5,27,28]. Several wild-type subjects and 10 α-thal carriers of known deletions (−α^3.7^; −α^4.2^; –(α)^20.5^; –MED; and –CAL) were used as controls. MLPA analysis was used for identification of rearrangement (new or rare deletions or duplications) in the α- and β-globin gene cluster. Ligation and amplification were performed on the ABI 2720 Thermal Cycler (Applied Biosystems, Foster City, CA, USA). The products were separated by capillary electrophoresis on the ABI-3130XL Genetic Analyzer (Applied Biosystems, Foster City, CA, USA). The quantitative analysis was carried out with the Coffalyser software (MRC-Holland, Amsterdam, The Netherlands). The result of the MLPA was obtained by comparing the values of samples under study with a pool of normal subjects.

### 4.4. qRT-PCR

A Quantitative Real Time PCR (qRT-PCR) was performed both with the Power SYBR-Green PCR-Master mix in the ABI 7900HT System (Applied Biosystems, Foster City, CA, USA) [27,28] and with the SsoAdvanced Universal SYBR Green Supermix (Bio-Rad, Hercules, CA, USA) in the CFX Connect Real-time PCR Detection System (Bio-Rad, Hercules, CA, USA), following the manufacturer’s instructions [29]. The primers for the quantitative analysis of 33 region alongside the α-globin gene cluster, chosen outside repeated sequences, reported in Table S1, were designed using the “Primer Express” software v.3.0 (Applied Biosystems, Warrington, UK) and the Primer-BLAST program available at http://www.ncbi.nlm.nih.gov/tools/primer-blast/ (accessed on 12 September 2022) [30], respectively. All samples were analyzed on triplicate, using 15 ng of genomic DNA. The beta-2-microglobulin (*B2M*), present on chromosome 15, was used as reference gene.

### 4.5. Breakpoint Characterization

The definition of breakpoint sequences was analyzed by long-range gap-PCR using a TripleMasterPCR System (Eppendorf, Hamburg, Germany) with primers shown in Table S2. The UCSC browser was used for the representation of the deletions on chromosome 16 [31,32].

## 5. Conclusions

In conclusion, the combination of multiple approaches is a reliable method through which we identified, in families or single patients from Southern Italy, five new α-thalassemia deletions: the --Sciacca, --FG, --PA, --Puglia, and –AG, ranging from about 7 kb to 265 kb. The identification of five new α-thal deletions highlighted the extreme heterogeneity of the molecular basis of α-thalassemia in Southern Italy.

As has already occurred for other point mutations identified by us, such as Hb Policoro and Hb Bernalda [25,26], it is likely that these five new deletions could be identified in other countries of the Mediterranean basin as already occurred for --CAL --MED, and --MED II [2,9,12,19].

The analysis of the regions involved in the breakpoints confirmed that Alu repetitive sequences are frequently involved in homologous and non-homologous recombination events in the α-globin gene cluster [17].

The high number of new α^0^-thal deletions identified by us suggest that we pay attention to the negative samples for the most common α-thal deletions, which should be analyzed using appropriate assays such as MLPA, qRT-PCR, or Next Generation Sequencing (NGS) with long reads to prevent Hb H Disease or hydrops fetalis.

## Figures and Tables

**Table 1:** Hematologic, biochemical data, and α-genotype of the families with the new **α**-thalassemia deletions.

Families (no./Relationship)		Sex	RBC (×10^12^/L)	Hb (g/dL)	Ht (%)	MCV (fL)	MCH (pg)	MCHC (L/L)	Serum Iron (mg/dL)	Ferritin (ng/mL)	Bilirubin Tot (mg/dL)	Bilirubin Ind (mg/dL)	Hb A2 (%)	Hb F (%)	Hb H (%)	α Genotype
1	I.1	M	5.55	12.3	39.3	70.8	22.2	31.3	=	=	=	=	2.5	1.9	=	--Sciacca/αα
2	I.1	M	5.68	15.6	45.9	80.8	27.5	34.0	57	168	0.68	0.53	2.2	=	=	-α3.7/αα
	I.2	F	6.15	13.0	41.0	66.6	21.1	31.6	31	12	=	=	2.3	0.7	=	--FG/αα
	II.1	M	6.18	11.6	39.4	63.7	18.8	29.5	67	154	=	=	1.1	0.5	6.7	-α3.7/--FG
	II.2	M	6.02	11.4	38.7	64.2	19.0	29.5	111	172	=	=	1.2	=	9.9	-α3.7/--FG
	II.3	F	4.98	9.1	30.1	60.5	18.4	30.4	66	132	=	=	1.2	0.6	7.6	-α3.7/--FG
3	I.1	F	4.01	12.6	37.7	94.0	31.6	33.6	=	142	0.44	0.31	2.5	0.5	=	αα/αα
	II.1	M	6.78	14.1	45.4	67.0	20.9	31.1	=	555	0.72	0.62	2.4	0.4	=	--PA/αα
	III.1	F	5.99	12.0	37.7	63.0	20.1	31.8	=	19	0.46	0.36	2.5	0.7	=	--PA/αα
	III.2	M	5.39	10.9	34.4	63.8	20.3	31.8	=	49	0.68	0.54	2.6	0.4	=	--PA/αα
4	I.1	M	6.28	15.7	43.9	70.0	25.0	35.7	=	56	0.49	0.34	2.6	=	=	--PA/αα
	II.1 *	M	5.00	14.8	35.2	70.0	29.5	41.9	=	121	0.64	0.49	6.3	=	=	--PA/αα
	II.2	M	3.88	16.4	35.5	91.0	42.3	46.2	=	186	0.87	0.66	3.0	=	=	αα/αα
5	I.1	F	6.09	13.9	44.6	73.1	22.8	31.2	78	134	0.36	0.27	2.7	0.3	=	--Puglia/αα
6	I.1	M	6.00	13.6	42.5	70.8	22.7	32.0	=	69	=	=	2.8	=	=	--AG/αα
	I.2	F	4.10	12.5	38.2	93.2	30.5	32.7	=	20	=	=	3	=	=	αα/αα
	II.1	F	4.97	10.5	33.8	68.0	21.1	31.1	=	22	=	=	2.5	=	=	--AG/αα
	II.2	M	6.71	14.1	44.4	66.2	21.0	31.8	=	43	=	=	2.5	=	=	--AG/αα

* β genotype: β IVS-II-745 (C>G)/β.

## Data Availability

All relevant data have been reported within the manuscript and its supporting information files.

## Supplementary Materials

The following supporting information can be downloaded at: https://www.mdpi.com/article/10.3390/ijms24032577/s1.
