# Supplementary material for: Alpha-Thalassemia in Southern Italy: Characterization of Five New Deletions Removing the Alpha-Globin Gene Cluster
Source: Int J Mol Sci. 2023 Jan 30;24(3):2577. doi: 10.3390/ijms24032577 (PMC9916800; doi:10.3390/ijms24032577)
Supplement: Supplementary file 1 [file ijms-24-02577-s001.zip › ijms-2150846-supplementary.pdf]

International Journal of
Molecular Sciences

MDPI

Alpha-thalassemia in Southern Italy: characterization of five new deletions removing the alpha-globin gene cluster

Giovanna Cardiero [1], Gennaro Musollino [1], Romeo Prezioso [1], Vincenzo Nigro [2,3], Giuseppina Lacerra [1,*]

[1] Istituto di Genetica e Biofisica “Adriano Buzzati Traverso” (IGB-CNR), Consiglio Nazionale delle Ricerche, 80125 Napoli,
[2] Department of Precision Medicine, University of Campania L. Vanvitelli, Naples, Italy,
[3] Telethon Institute of Genetics and Medicine (TIGEM), Pozzuoli (NA, Italy),
* Correspondence: giuseppina.lacerra@igb.cnr.it

Supplementary material

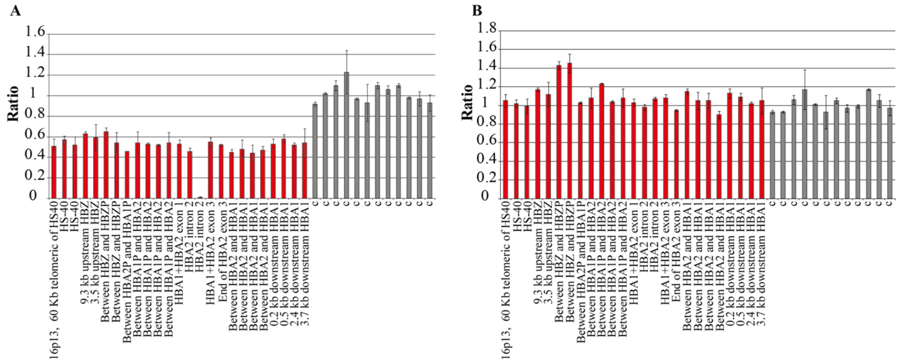

Figure S1. MLPA pattern of the family 6 with the deletion --AG. A) The father (αα/--AG) show the reduction of level of all the 25 MLPA probed; **B**) the mother showed the duplication of the zeta gene; The son (Fig 2E), compound heterozygotes for the --AG deletion and for the duplication of the zeta gene, showed the deletion of 23/25 MLPA probes and normal level of the 6 and 7 probes.

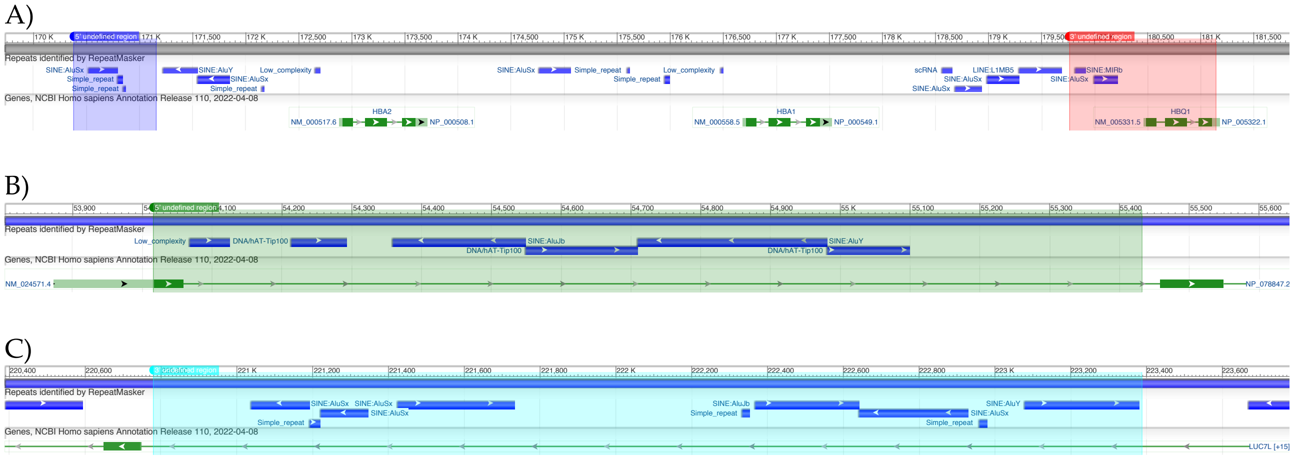

Figure S2. Repetitive elements present in the 5′ and 3′ undefined regions of the - - Puglia and - - Sciacca α-thalassemia deletions. A) The 5′ and 3′ undefined region of the --Sciacca contain respectively the following repeats: in 5′ SINE: AluSX and 2 Simple repeats; in the 3′ SINE: MIRb and SINE: AluSx. The regions, where the breakpoints could be located, are highlighted. **B**) The 5′ undefined region of the -- Puglia contains the following repeats: low complexity, DNA/hAT-Tip 100, SINE: AluJb, DNA/hAT-Tip 100, SINE: Alu Y, DNA/hAT-Tip 100. **C**) The 3′ undefined region of the --Puglia contains the following repeat: SINE: AluSX, Simple Repeat, SINE: AluSX, SINE: AluSX, Simple Repeat, SINE: AluJb, SINE:AluSX, Simple Repeat, SINE: AluY.

Table S1. Primers and positions of amplicons for quantitative real time PCR. Primer positions are according to reference sequence NC_000016.10 and NC_000015.10.

n.	Oligo forward	Position on Chr 16	bp	n.	Oligo reverse	Position on Chr 16	bp	Fragments length (bp)
1-for	GGGAATCTGGGAGACCTGTGA	48298-48318	20	1-rev	CCTTCAGCTGGATTTGAGAAGAAC	48379-48356	23	82
2-for	CAGTGGCATCAACAGCCTCAT	51495-51515	20	2-rev	ACTCTACTGCAGGTAAGAGGGAAAACT	51569-51543	26	75
3-for	AAGAGGAAGATGAGGACGAAGAAGA	53943-53967	24	3-rev	GCCAGACCCTCCTGGAACA	54015-53997	18	73
4-for	CCACTTCTGCCCTTGGTCTTT	55433-55453	20	4-rev	CCTGGCCGTATTCTAGGGCTAT	55507-55486	21	75
5-for	TTTGACAGCTGGCTGGTTGA	84303-84322	19	5-rev	GGGCGAGCACTAGTGGACAA	84378-84359	19	76
6-for	CTGGCCCATAAGAAGGAGGTTAA	110480-110502	22	6-rev	GGGACCTCAAGGAAGCTTATGAC	110549-110527	22	70
7-for	CAGTGTGGCAGCGTTGAGA	146054-146073	19	7-rev	CCATGACAACTCATTAATCCATTCC	146146-146122	24	93
8-for	CCAGACAGACACAAATGAGAGCAT	148063-148086	23	8-rev	CTTTGGTTGTTTTTGCCTGCTT	148138-148117	21	76
9-for	TCCCCTGCATCCCTTTCAG	171090-171109	19	9-rev	TAGTAATAATCAGTGAGACTGTGGAATGG	171157-171129	28	68
10-for	GCACCGTGCTGACCTCCAAA	173572-173591	19	10-rev	CCCATCGGGCAGGAGGAA	173637-173620	17	66
11-for	CCTCTGCCTGCGTTTGTGAT	178415-178434	19	11-rev	AGACACCGTCTTCCTGACAGTTG	178486-178464	22	72
12-for	CGACCTTCCCCGTGTTTG	181140-181159	19	12-rev	CGAGAGAGCACGGCAAGAA	181201-181183	18	62
13-for	GTGGACACCCTCCTGGGATT	183212-183231	20	13-rev	TCTGCACCTCTGGGTAGGTTCT	183359-183338	22	148
14-for	GGCCGAGGACACTGATGCT	185599-185618	19	14-rev	CATCCTGTCCCCCGTGTCT	185673-185655	18	75
15-for	GTGCCCTGCCATCTACTGGAT	188850-188870	20	15-rev	CACCAGCACCTCACAGAAACC	188926-188906	20	77
16-for	CCACAAGGGCACGGAAAGTA	195009-195028	19	16-rev	CAGGATGAGCATGGGTGAAAGT	195084-195063	21	76
17-for	AAGTGTAACTTGCCACCGAAGTG	199102-199124	22	17-rev	GCCTACCTTGGTCTCCATGACA	199166-199145	21	65
18-for	GCATCCTTACTGCCAGCATTG	206281-206301	20	18-rev	ACAAAGGTGCCACTGGTTTCAT	206355-206334	21	75
19-for	CACAGGCCGGTGGTCACT	211889-211908	19	19-rev	CAGCACAAGGGCACCTGTCT	211969-211950	19	81
20-for	AGGGCCAAGTCGTGGATTTT	220704-220723	19	20-rev	TGAGGTCTGCATTTTCTGTTTCTTT	220779-220755	24	76
21-for	AGCACGTGGCATACTCACTAACAAA	223389-223413	24	21-rev	CACCATACAAGGCAGACCCGT	223483-223463	20	95
22-for	CACTGAGGATAGGCTTGGGTTCT	224913-224935	22	22-rev	CCAGGGGAATGGCCAGTAGTAT	225011-224990	21	99
23-for	TGTGACTCACGGCTCGATGA	228323-228342	20	23-rev	TCAGTGTATCTTTGCCTGCCTACA	228400-228377	24	78
24-for	GGCGGGACCTATGGAAAGAA	231423-231442	19	24-rev	CGTGTGGTACACGTACAAATATGACA	231551-231526	25	129
25-for	GCCGAGGTTGCTCGTCTGT	235383-235402	19	25-rev	CCCAGTGTTTTCCTCCCATCTAG	235458-235436	22	76
26-for	ACACAGTAGCTGTCCTTGTCTGACA	240006-240030	24	26-rev	AGACACCCACAACAGCCAATG	240080-240060	20	75
27-for	GCTGTTGATGAGCAAATCTTTGG	243523-243545	22	27-rev	CAGGCAGTTCATGATTCAACCA	243597-243576	21	75
28-for	GGTTCAGGTGGGTGCTCATCT	247986-248006	20	28-rev	CAGAAGGCAAAGCCCCATTAC	248061-248041	20	76
29-for	GAGGCTCAGCCCTGTGATCTT	260247-260267	20	29-rev	GCAGCCTCACTTGGAGGTTCT	260321-260301	20	75
30-for	TGATTGGCAGAAACGCAACTAA	273302-273323	21	30-rev	CACCATTTTGAGCCTGTGGAA	273395-273375	20	94
31-for	AGCGTCCATCACTGCTTTCTG	285655-285675	20	31-rev	TCCCCTTCCATTATGAGCAGTCT	285735-285713	22	81
32-for	CAGGGCTCCTATCTCAGGAAGAC	289920-289942	22	32-rev	GCACAGTTCTGTTTGTCCAATCC	290002-289980	22	83
33-for	GGCCTCCTGCCTGTTGCT	297604-297623	19	33-rev	GCCAGAACCTCCTCATAAAACTCA	297662-297639	23	59
		**Chr 15**				**Chr 15**		
B2M	TCTGCTGCGGCTCTGCTTC	44712081-44712099	19		GGTGCTAGGACATGCGAACTTAG	44712161-44712139	23	80

B2M: β2 microglobulin

Table S2: Primers for the long-range PCR for the definition of breakpoints of the new deletions.

Primer name	Sequence	Position on chr 16	PCR length (bp)
**Long-range PCR for the --(PA)**			
7-for	5′-CAGTGTGGCAGCGTTGAGA-3′	146054-146072	501
A-rev	5′-GCGCTCAATCTCCGACAGCTCCGA-3′	180344-180321	
**Long-range PCR for the --(AG)**			
B-for	5′-TATGGATCCCTAACCCTGACCCTAACCC-3′	telomeric	522
C-rev	5′-GGCCTGACCTACCTTCTTGAAG-3′	285027-285006	
**Long-range PCR for the --(FG)**			
D-for	5′-GCTGAGGGAACACAGCTACATCTACA-3′	170978-171003	~2.600
14-rev	5′-CATCCTGTCCCCCGTGTCT-3′	185673-185655	
9-for	5′-TCCCCTGCATCCCTTTCAG-3′	171090-171108	~2.600
14-rev	5′-CATCCTGTCCCCCGTGTCT-3′	185673-185655	
9-for	5′-TCCCCTGCATCCCTTTCAG-3′	171090-171108	~1.400
E-rev	5′-CCACCTTTGCCTCCTGCAC-3′	184453-184435	
